# Loss of periodontal ligament fibroblasts by RIPK3-MLKL-mediated necroptosis in the progress of chronic periodontitis

**DOI:** 10.1038/s41598-019-39721-1

**Published:** 2019-02-27

**Authors:** J. Shi, J. Li, W. Su, S. Zhao, H. Li, L. Lei

**Affiliations:** 1 Nanjing Stomatological Hospital, Medical School of Nanjing University, Nanjing, Jiangsu 210008 China; 2 Central Laboratory of Nanjing Stomatology Hospital, Nanjing, Jiangsu 210008 China

## Abstract

Periodontal homeostasis is maintained by the dynamic equilibrium between cell death, differentiation and proliferation of resident cells in the periodontal microenvironment. Loss of resident periodontal ligament fibroblasts (PDLFs) has been a major challenge in the periodontal treatment. This study aimed to investigate the exact role of necroptotic cell death in periodontal diseases. Elevated levels of receptor-interacting protein serine-threonine kinases -1 (RIPK1), phosphorylated RIPK3, mixed lineage kinase domain-like protein (MLKL), phosphorylated MLKL and FLIP_L_ were observed in gingival tissues collected from patients with untreated chronic periodontitis; whereas no difference in caspase 8 was observed between the periodontitis and healthy control group. In contrast to the high incidence of necroptotic cell death in monocytes during live *P. gingivalis* infection with a low multiplicity of infection (MOI), necroptosis was only observed in PDLFs with a high MOI. Priming PDLFs with frozen thawed monocytes enhanced proinflammatory responses to *P. gingivalis* infection; moreover, frozen thawed monocytes stimulation triggered RIPK1, RIPK3 and MLKL-mediated-necroptotic cell death in PDLFs. These results indicated that RIPK3 and MLKL-mediated-necroptotic cell death participated in the pathogenesis of periodontitis, and DAMPs released from monocytes after *P. gingivalis* stimulation by necroptosis triggered not only inflammatory responses, but also necroptosis of PDLFs.

## Introduction

Periodontitis, an inflammatory disease that affects the supporting tissues of the teeth, is initiated by the dysbiosis of dental biofilms in the periodontal milieu. Several putative periodontal pathogens, such as *Porphyromonas gingivalis, Tannerella forsythia* and *Prevotella intermedia*, have been implicated in the process of periodontal disease development^1^. In addition, periodontal disease results not from individual pathogens but rather from polymicrobial synergy and dysbiosis, which perturbs the ecologically balanced biofilm associated with periodontal tissue homeostasis^2^. Bacterial cell wall components and intracellular contents can stimulate the host immune system, leading to the production and release of pro-inflammatory mediators^3^. Matrix metalloproteinases (MMPs), proteolytic enzymes that are responsible for the degradation of the organic extracellular matrix (ECM), have been reported to be associated with periodontal tissue destruction and alveolar bone absorption^4,5^. In addition, osteoclasts release HCl through proton pumps on the cell membrane into bone resorption pits, leading to the dissolution of crystalline hydroxyapatite, the main inorganic component of the periodontium^6^.

Although the destruction of the organic and inorganic extracellular contents of the periodontium has been widely investigated, the loss of the cellular components of the periodontal tissue has been less well studied. Over the last decade, several newly reported regulated cell deaths (RCDs), such as necroptosis, pyroptosis, and NETosis (cell death associated with the release of neutrophil extracellular traps (NETs)), have been found to play a key role in the pathogenesis of inflammatory diseases^7^. Pyroptosis, which is characterized by the formation of the inflammasome complex, including the nucleotide-binding oligomerization domain-like receptor containing pyrin (NLRP), apoptosis-associated speck-like protein containing a CARD (ASC) and caspase-1, has been reported in diseased periodontal tissue^8^. In contrast to the pro-inflammatory pyroptosis, with its release of large amounts of death-associated molecular patterns (DAMPs), the process of NETosis is similar to that of apoptosis, with less capability to promote inflammatory cytokine secretion while promoting bacterial clearance^9^.

Necroptosis, mediated by receptor-interacting protein serine-threonine kinases-3 (RIPK3) and its substrate, mixed lineage kinase domain-like protein (MLKL), is a recently characterized form of regulated necrosis that contributes to the development of non-pathogen-related inflammatory diseases, such as intestinal inflammation^10^, ischemia–reperfusion injury (IRI) in the brain^11^ and cholestasis involving liver injury and inflammation^12^. With its profuse release of DAMPs such as alarmins, mitochondria, ribosomes and DNA into the extracellular environment, the execution of necroptosis may generate robust pro-inflammatory responses, leading to the destruction of local tissue^13^.

Previous study has demonstrated that less apoptosis is observed in the inflamed periodontal tissue^14^. Such phenomenon raises the question that how the principal resident cells, periodontal ligament fibroblasts (PDLFs), are lost during the progress of periodontal diseases. As periodontal homeostasis is maintained by a dynamic equilibrium between the cell death, proliferation and differentiation of resident cells in the periodontal microenvironment, the exact mechanism of loss of PDLFs remains to be unclear. Therefore, the purpose of the study was to investigate the role of necroptosis in human periodontal disease progression and the role of necroptosis in the cell death of PDLFs.

## Results

### Increased Expression of Necroptosis-related Genes in Gingival Tissues

**Figure 1:**
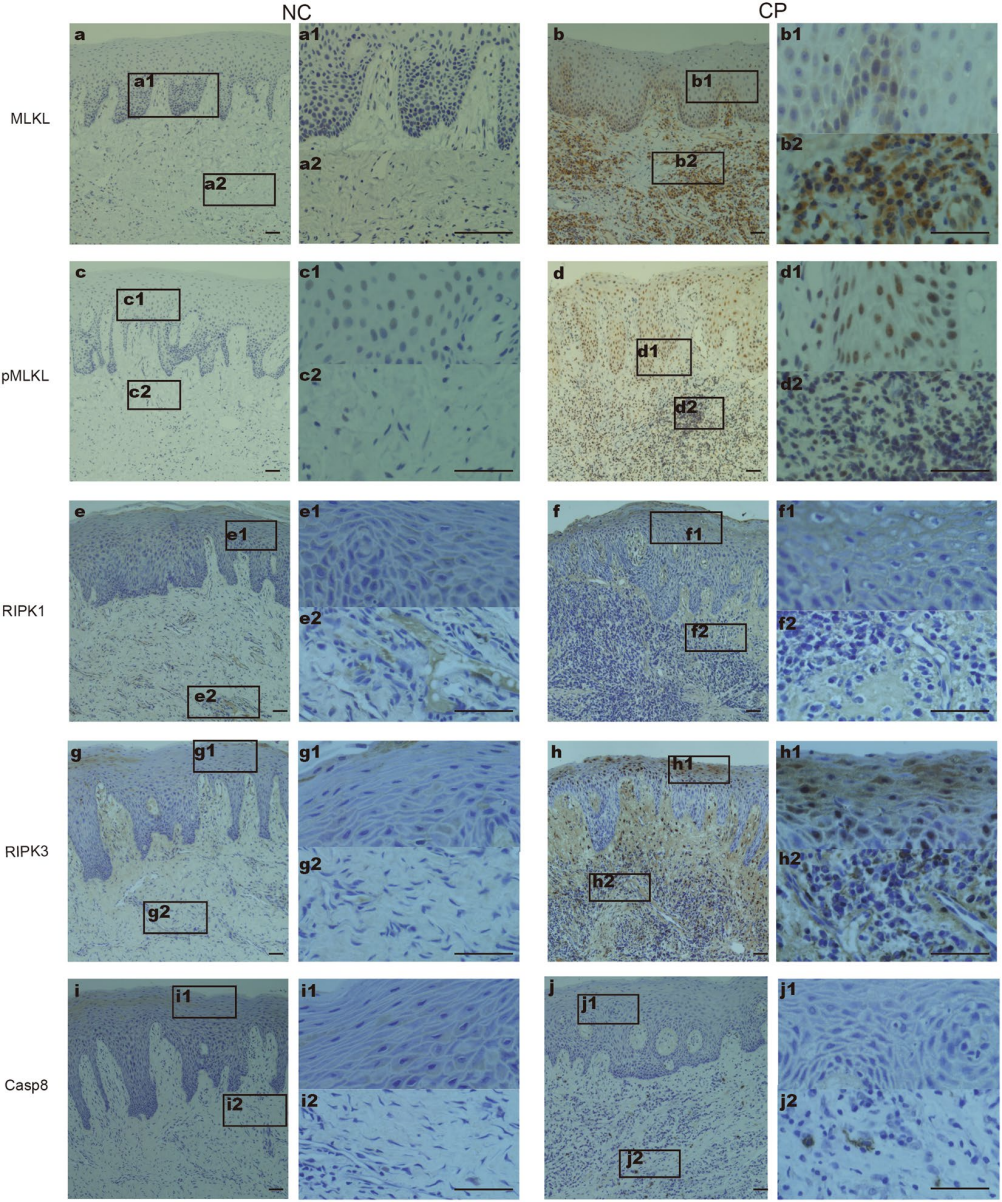
Expression of necroptosis-related genes in periodontitis periodontal tissues. Samples from normal control (NC) (n = 18) and chronic periodontitis patients (CP) (n = 14) were stained for MLKL (**a,b**), pMLKL (**c,d**), RIPK1 (**e,f**), RIPK3 (**g**,**h**) and caspase-8 (**i,j)**. Figures (a1/2–j1/2) show higher magnifications of the same sections in Figures (**a–j**). The scale bar represents 50 µm.

MLKL dependent necroptosis has been demonstrated to participate in the progression of periodontitis in experimental mouse models^15^, while compelling evidence supporting that necroptosis is active in human periodontitis is still absent. We supposed that necroptosis is activated in advanced periodontitis in humans, so we first utilized immunohistochemistry to explore necroptosis-related gene expression in the gingiva. Low levels of MLKL and pMLKL were observed in the normal control (Fig. 1a,c), whereas widespread expression was detected in both the gingival epithelia and connective tissues from chronic periodontitis patients (Fig. 1b,d), indicating the presence of necroptosis during periodontitis progression. The expression of pMLKL could be detected in whole layers of the epithelium, while MLKL was mainly in the basal layer. Elevated levels of RIPK1 and RIPK3 were observed in inflamed gingiva and showed moderate positive staining in both the epithelia and connective tissues (Fig. 1e–h). As caspase 8 is required for cells driving towards apoptosis or necroptosis^16^, so we further investigated changes of caspase 8 in the inflamed gingiva. Interestingly, no significant difference in the expression of caspase-8 was observed between normal and inflamed periodontal tissues (Fig. 1i,j). The semi-quantitative data are presented in Table 1.

**Table 1:** Semi-quantitative results (mean ± SD) of immunostaining for MLKL, pMLKL, RIPK1, RIPK3, Caspase-8 in the normal and periodontitis group.

	Normal control (n = 18)	Periodontitis (n = 14)
MLKL	0.5556 ± 0.1506	2.643 ± 0.3164****
pMLKL	0.2222 ± 0.08306	2.643 ± 0.4174****
RIPK1	2.306 ± 0.2144	3.25 ± 0.1015**
RIPK3	2.139 ± 0.2093	3 ± 0.2514*
Caspase-8	0.6944 ± 0.1624	0.8929 ± 0.07736

^*^p < 0.05; **p < 0.01,***p < 0.005, ****p < 0.0001.

### mRNA Transcription in Gingival Tissues

**Figure 2:**
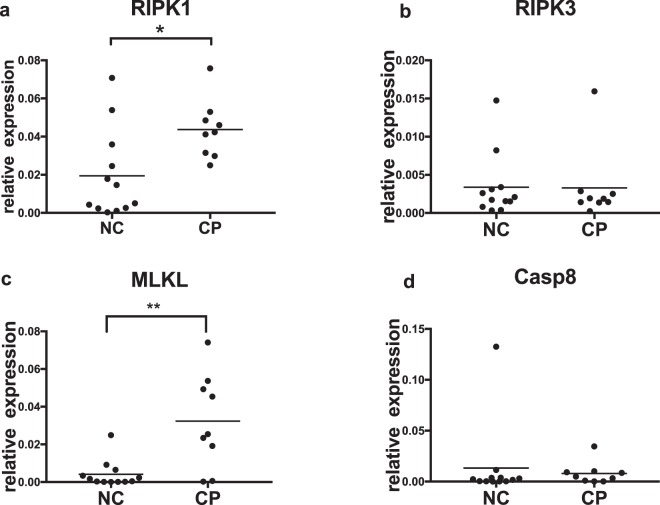
Transcription of necroptosis-related genes in periodontal tissues. Total RNA was extracted from normal gingival tissues (NC) (n = 12) and tissues with chronic periodontitis (CP) (n = 9). Levels of gene transcription were normalized to that of the internal control, β-actin. (*p < 0.05; **p < 0.01).

To confirm the immunohistological findings, we next explored necroptosis-related gene transcription in gingival tissues. The level of MLKL in the tissues with periodontitis was almost eight-fold higher than that in normal controls (Fig. 2a). Similarly, RIPK1 also showed prominent upregulation in tissues with periodontitis compared with normal control samples (Fig. 2c). However, we did not observe differences in the transcription of RIPK3 or caspase8 (Fig. 2b,d).

### Immunoblotting for Necroptotic Molecules in Gingival Tissues

**Figure 3:**
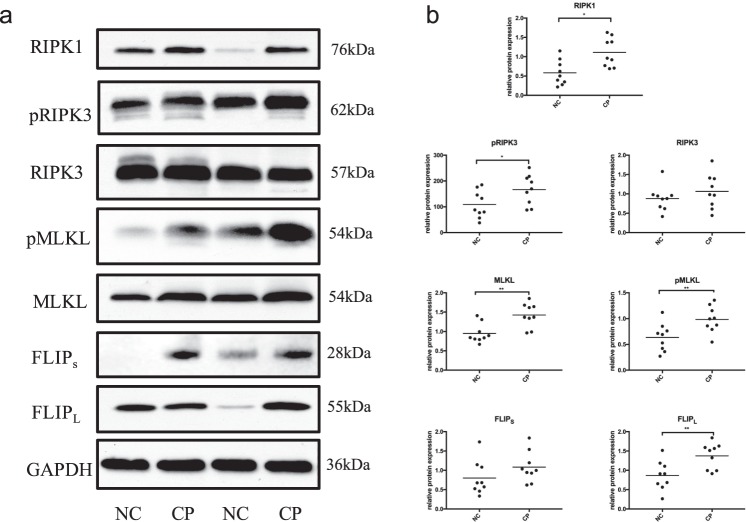
Expression of necroptosis pathway components in gingival tissues. (**a**) Gingival tissues from normal control (NC) (n = 9) and chronic periodontitis (CP) (n = 9) patients were analyzed by immunoblotting. All samples were treated at the same time and the gels were processed in parallel. The images were collected from different gels with the same loading quantity of protein samples and were the representative of four experiments. (**b**) Levels of necroptotic genes were normalized to those of the internal control GAPDH.

Samples from 9 subjects with a healthy periodontium and 9 periodontitis patients were analyzed by immunoblotting. The protein levels of RIPK1, RIPK3, phosphorylated RIPK3, MLKL, phosphorylated MLKL, FADD-like intedeukin-1-β converting enzyme inhibitory protein (c-FLIP) and GAPDH in the gingival samples were exhibited in Fig. 3a. Semiquantitative Western blot analysis showed that RIPK1, pRIPK3, MLKL, pMLKL and FLIP_L_ levels were significantly higher in the chronic periodontitis group than in the normal controls (Fig. 3b). Although RIPK3 and FLIP_s_ tended to increase in inflamed gingiva, no difference was found between the two groups.

### Activation of Necroptosis in *P. gingivalis*-treated Human PDLFs

**Figure 4:**
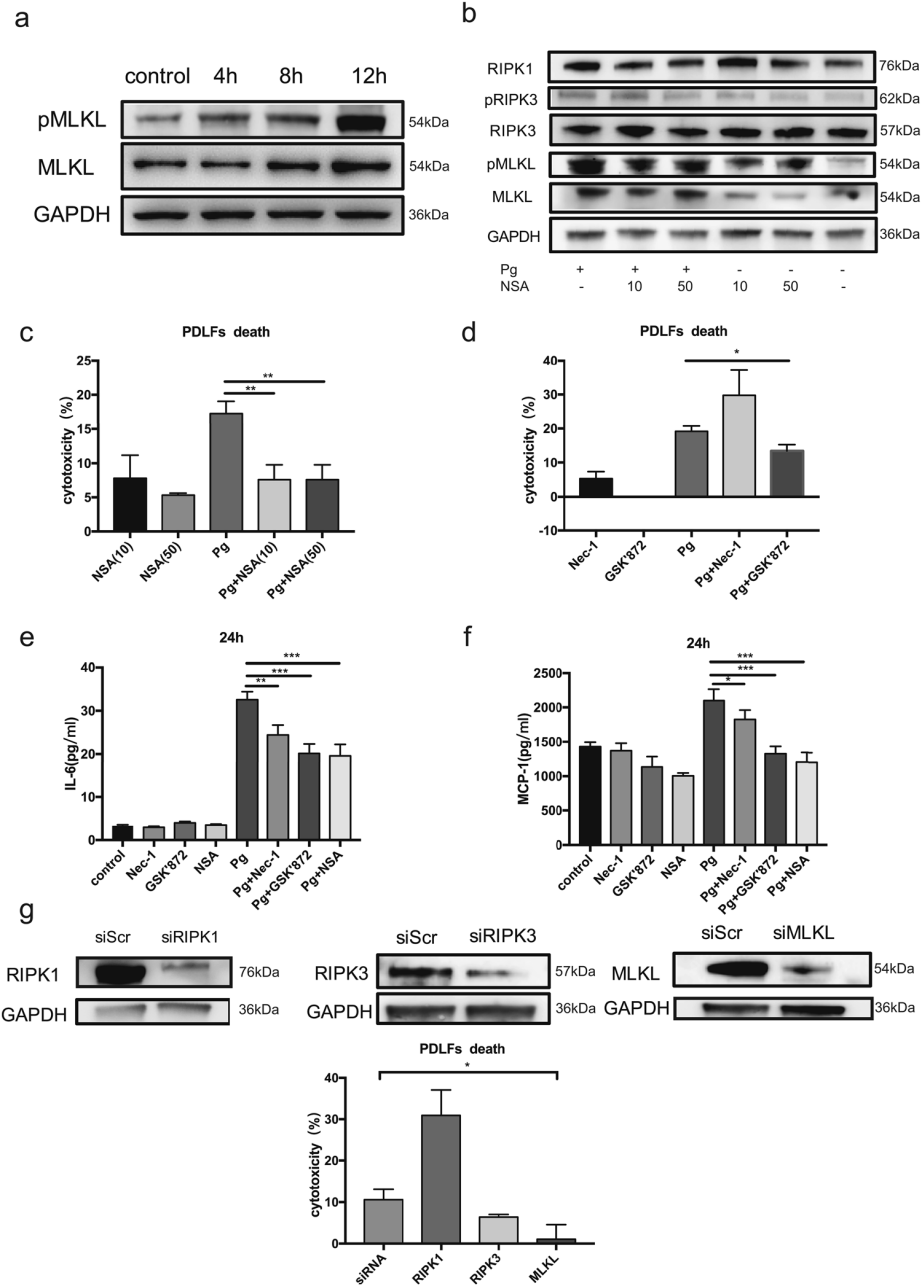
MLKL mediated the necroptosis elicited by *P. gingivalis* in periodontal ligament fibroblasts. (**a**) Expression of MLKL and pMLKL in the lysates of PDLFs after *P. gingivalis* infection (MOI = 400). (**b**) Effects of NSA on MLKL, pMLKL, RIPK1, RIPK3 and pRIPK3 at 4 h. The images were collected from different gels with the same loading quantity of protein samples in both figures a and b. (**c**,**d**) Cell death by release of LDH. (**e,f**) IL-6 and MCP-1 levels, as shown by ELISA. (**g**) Cell death after gene knockdown; The images were collected from the same gel. (*p < 0.05; **p < 0.01; ***p < 0.001).

As a high positive detection rate of *P. gingivalis* in deep periodontal pockets was reported in Chinese subjects, varying from 62.5% to 92.5% depending on the probing depth using species-specific DNA Probe^17^. To further understand how the cell components were lost during periodontitis progression, we infected PDLFs with the periodontal pathogen *P. gingivalis*. MOI of 10 and 50 failed to induce significant cell death in PDLFs, whereas higher MOI of 100 and 400 resulted in significant incidence of cell death in PDLFs. pMLKL, a marker of execution of necroptosis, was clearly observed when the MOI reached 400, while lower MOIs failed to induce significant pMLKL expression. Enhanced levels of pMLKL and MLKL can be observed at 4 h, and prominent expression occurs at 12 h (Fig. 4a). NSA, a specific inhibitor of MLKL, blocked the upregulation of RIPK1, pRIPK3, MLKL and pMLKL after *P. gingivalis* infection, while the levels of RIPK3 were not altered after NSA and *P. gingivalis* treatment (Fig. 4b). In line with the decrease in pMLKL and MLKL by NSA treatment, we found that NSA at both 10 μM and 50 μM effectively suppressed cell death in PDLFs, as shown by the levels of LDH in the supernatants (Fig. 4c). GSK’872 at 10 μM also decreased cell death in PDLFs. In contrast, pretreatment with Nec-1 to inhibit RIPK1 failed to reduce cell death after bacterial infection; moreover, cell death after Nec-1 incubation tended to increase (Fig. 4d). Furthermore, we explored the effects of NEC-1, GSK’872 and NSA on pro-inflammatory cytokines; Nec-1, GSK’872 and NSA treatment significantly reduced the levels of IL-6 and MCP-1 in the supernatants (Fig. 4e,f). The silencing of MLKL reduced the cell death rate caused by *P. gingivalis*, whereas RIPK1 silencing increased cell mortality, and RIPK3 knockdown did not significantly affect cell death (Fig. 4g).

### Activation of Necroptosis in *P. gingivalis*-treated Human PDLFs by DAMPs

**Figure 5:**
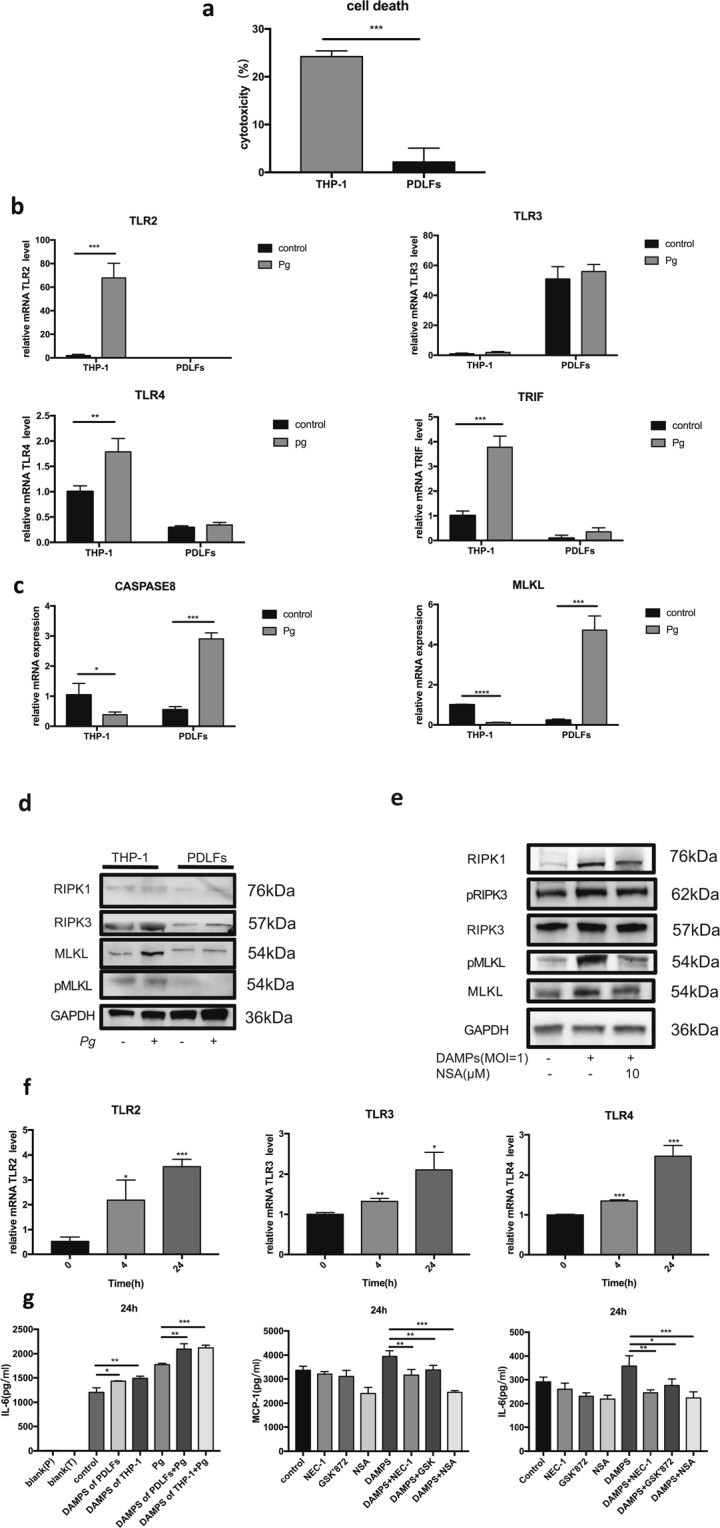
DAMPs from THP-1 cells induced further necroptosis and upregulated cytokine production in periodontal ligament fibroblasts. (**a**) Cell death after *P. gingivalis* infection (MOI = 100) in THP-1 cells and PDLFs at 4 h. (**b,c**) Transcription of TLR2, TLR3, TLR4, TRIF, caspase 8 and MLKL in *P. gingivalis* treated groups (MOI = 100) and control groups in THP-1 cells and PDLFs for 2 h. **(d**) The protein level of RIPK1, RIPK3, MLKL and pMLKL in THP-1 cells and PDLFs with or without *P. gingivalis* infection (MOI = 100) for 4 h. (**e**) The effects of DAMPs and NSA (10 μM) on the expression of MLKL, pMLKL, RIPK1, RIPK3 and pRIPK3. The images were collected from different gels with the same amount of samples in both figure d and e. (**f**) DAMPs from THP-1 cells upregulated the level of TLR2, TLR3 and TRIF in PDLFs at 4, 24 h. (**g**) DAMPs from THP-1 cells and PDLFs induced the production of IL-6 in the supernatant of PDLFs. The level of IL-6 was upregulated in PDLFs stimulated with *P. gingivalis* infection (MOI = 100) for 24 h with pretreatment with DAMPs from THP-1 cells and PDLFs for 24 h. Blank(P) and Blank(T) meant DAMPs in the same culture medium as control without cells. IL-6 and MCP-1 levels increased in the supernatant of PDLFs treated with DAMPs from THP-1 cells and decreased by pretreatement with Nec-1(50 μM), GSK’872(10 μM) and NSA(10 μM). (*p < 0.05; **p < 0.01; ***p < 0.001).

Although significant cell death was observed in PDLFs in a high MOI of 400 *P. gingivalis*, no obvious cell death was observed in a low MOI of bacteria. As we have previously reported that *P. gingivalis* can induce necroptosis in monocytes, we further compared the cell death of PDLFs and monocytes. As expected, in a MOI of 100, significant cell death was observed in monocytes, whereas less cell death was found in PDLFs (Fig. 5a). To explore the mechanism of such difference, we further investigated the expression of pattern recognition receptors, which inform the host of the invading danger of bacteria invasion. Monocytes displayed significant TLR2 and TLR4 expression, and PDLFs showed no obvious up-regulation. TRIF could bind to TLR3/TLR4 and interact with RIPK1, leading to necroptosis^18^. Enhanced transcription of TRIF was found in monocytes when compared to PDLFs (Fig. 5b). In addition, PDLFs showed significant higher upregulation of caspase-8 upon bacteria invasion; in contrast, monocytes demonstrated more transcription of MLKL (Fig. 5c).

Periodontitis could be ascribed to the host response to bacteria instead of the direct pathogenic effect. DAMPs, constantly released from dying cells, evoke sustained inflammation with the destruction of tissues and many diseases have been proved to be associated with DAMP-mediated inflammation such as autoimmunity and neurogenic disease^19,20^. As PDLFs demonstrated a low tendency to develop necroptotic cell death compared to monocytes during *P. gingivalis* infection (Fig. 5d), we further explored whether necroptotic cell death of monocytes could mediate cell death and pro-inflammatory response in the PDLFs. We utilized the frozen thawed monocytes to mimic DAMPs release of cell death after *P. gingivalis* infection in monocytes. PDLFs showed obvious cell death and necroptotic pathway related gene expression after stimulation with a MOI of frozen thawed monocytes. Significant increased RIPK1, pRIPK3, MLKL and pMLKL was observed in PDLFs after stimulation with DAMPs; in addition, NSA pretreatment significantly reduced RIPK1, pRIPK3, MLKL and pMLKL expression in PDLFs after DAMPs stimulation (Fig. 5e). Moreover, DAMPs stimulation induced significant release of MCP-1 and IL-6 in PDLFs. Furthermore, priming the PDLFs with DAMPs induced more expression of pattern recognition receptors and more proinflammatory cytokine release after *P. gingivalis* infection (Fig. 5g,f).

## Discussion

Cell death, which was previously believed to be the result of inflammation, has been implicated in the pathogenesis of inflammatory diseases^21^. Dying cells may release DAMPs, such as high-mobility group protein B1 (HMGB1), IL-33 and ATP, into the extracellular space^22^. High levels of HMGB1 have been reported in the gingival crevicular fluid and inflamed gingiva of periodontitis patients^23^. While less apoptosis was observed in inflamed periodontal tissue^14^, our present research for the first time observed that increased necroptosis was observed in the inflamed human periodontal tissue. In contrast to high tendency to undergo RIPK1/3-MLKL mediated necroptosis in monocytes in response to *P. gingivalis* infection^15,24^, PDLFs was rather resistant to necroptosis in response to live *P. gingivalis* infection, however, DAMPs released from monocytes triggered the necroptotic cell death of PDLFs, leading to loss of resident cells in the periodontal tissue.

Apoptosis and necroptosis have evolved as counterbalances in the first line of defense against inflammatory stimuli, with many molecules shared by both death pathways. In the death receptor pathway of apoptosis, the binding of death receptors on the cell surface leads to the formation of a death-inducing signaling complex (DISC), including FADD and caspase-8^25^. The binding of procaspase-8 to FADD prompts the recruitment of additional procaspase-8 molecules to form dimeric caspase-8, leading to the execution of apoptosis by cleaving dimeric caspase-3 and caspase-7. However, when the proapoptotic caspase-8 is inhibited or overwhelmed, the kinases RIPK1 and RIPK3 become phosphorylated, leading to the recruitment and activation of MLKL by pRIPK3^26,27^. In the progression of periodontitis, the levels of caspase-8 were not altered, whereas higher levels of cFLIP were found in the inflamed periodontal tissue and promoted both the formation of cFLIP_L_-caspase-8 heterodimers and the cleavage of RIPK1 and RIPK3, thereby blocking both apoptosis and canonical RIPK1-mediated necroptosis. Indeed, the inhibition of apoptosis was observed in inflamed gingiva^14^, while in our research, increased necroptosis was observed in inflamed gingival tissues. These contradictory data indicated that alternative or RIPK1-independent necroptosis plays a critical role in the development of periodontal disease. In the presence of TLR3/4 ligands (e.g., bacteria, LPS and lipoteichoic acid) or interferons, TLR3/4 binding recruits the adaptor molecule TRIF, which interacts with RIPK3 and MLKL through their homotypic RHIM domains^28^.

Homeostasis in the periodontal milieu is maintained by a dynamic equilibrium between host defense cells and pathogenic microbials. We have demonstrated that in an animal model of periodontitis via *P. gingivalis* infection, the inhibition of MLKL by NSA helps reduce periodontal destruction^15^. In contrast, our present research found that RIPK-1 knockdown failed to suppress cell death; the release of LDH even increased after RIPK-1 knockdown. It must be noted that RIPK-1 has been recognized as a central controller in pro-survival/inflammatory NF-κB activation, apoptosis and necroptosis^29^. Therefore, a defect in NF-κB activation/cell survival after RIPK1/3 inhibition may partly account for the increased cell death in PDLFs. Our results were in line with previous reports that RIPK1 protects hepatocytes from TNF-induced death^30,31^.

Necroptotic cell death signaling contributes to the immune response to several infections by bacteria such as *Staphylococcus aureus*, *Streptococcus pneumoniae*, *Listeria monocytogenes* and *Escherichia coli*^32,33^. Currently, the role of necroptosis and other forms of cell death in bacterial infection is not clear. *P. gingivalis* encodes gingipain adhesin peptide A44, which hijacks the host’s clathrin-dependent endocytosis system, translocates to host mitochondria and generates an antiapoptotic effect^34^. The inhibition of host cell death may allow the intracellular persistence of live bacteria; therefore, the RIPK3/MLKL-dependent necroptosis of infected cells may help eliminate the cellular space necessary for intracellular bacterial survival, which can also lead to tissue damage.

Interestingly, PDLFs showed a lower tendency to develop necroptosis under the stimulation of *P. gingivalis* when compared with monocytes. This effect stressed that at the first line of defense against *P. gingivalis* invasion, sentinel cells such as monocytes detect danger signals and undergo necroptosis once the alerting signal mounts to the threshold for cell death^35^. It has been reported that DAMPs, acting as endogenous danger signal molecules, could be distinguished by gingival epithelial cells, leading to the disruption of epithelial barrier^36^. In consistent with previous results, DAMPs released from necroptotic monocytes induced the death of PDLFs and amplified the inflammatory reaction through enhancing the expression of toll like receptors. Although PDLFs have a low tendency to undergo necroptosis during live *P. gingivalis* infection, priming PDLFs with DAMPs from monocytes enhanced both proinflammatory response and cell death of PDLFs. Therefore, strategies targeting inhibition of necroptosis may reduce cell death of immune cells, thereby decrease DAMPs release and stop ‘vicious circle’ in the periodontal microenvironment.

It must be noted that *P*. *gingivalis* was able to maintain steady-state growth in environments containing lower oxygen levels (3% and 6%), while live *P. gingivalis* may undergo increased bacteria death with the prolonging of exposure to normoxia^37^. Therefore, cells-bacteria co-culture *in vitro* may not mimic the environment *in vivo*. In addition, the anaerobic *P. gingivalis* utilize various virulence factors, including fimbriae, lipopolysaccharide, hemagglutinins, serine phosphatase, and especially gingipains, to invade the host cells and to subvert the immune system^38^. Further experiments are needed to identify the virulent factors of different periodontal pathogens that induce necroptosis. Moreover, pyroptosis has been also found in the diseased periodontal tissue, and NLRP6 can induce pyroptosis by activation of caspase-1 in gingival fibroblasts^39^. In addition, we found a large discrepancy in the Caspase-8 level in the gingival biopsy of healthy control group; as apoptosis is an integral phenomenon in the tissue growth, exploring the occurrence of cell death modes in different situations, such as young and old subjects, may further improve our knowledge regarding the physiological and pathological role of apoptosis, pyroptosis, and necroptosis in maintaining the homeostasis of periodontal microenvironment.

In summary, RIPK1/3-MLKL-dependent necroptosis was involved in the progression of periodontitis, and the *P. gingivalis*-induced death of immune cells in the periodontal milieu not only led to DAMP-induced tissue damage but also to the further loss of periodontal stem cells in the periodontium. The use of inhibitors of the necroptotic pathway, especially MLKL, might help in the host modulation therapy of chronic periodontitis.

## Materials and Methods

### Study Population and the Collection of Tissue Samples

The study population was divided into two groups: (1) patients with a healthy periodontium and (2) patients with untreated chronic advanced periodontitis. Patients regarded as healthy controls had no signs of loss of supporting tissues, probing depths <4 mm and BOP sites less than 30%. Normal gingival tissues were negative BOP at sampling sites and taken during coronal lengthening, wisdom tooth extraction or orthodontic crown exposure of impacted teeth. Patients diagnosed with chronic advanced periodontitis had bone loss and attachment loss confirmed by radiographic evidence and had at least six teeth with probing depths >6 mm. Samples of inflamed gingival tissue were taken during the extraction of hopeless teeth with the mobility up to three degrees from patients with advanced periodontitis; sampling sites in the periodontitis group had severe alveolar bone loss (above 2/3), reduced attachment level, increase probing depths (>6 mm) and positive BOP. Participants were required to comply with the following systematic exclusion criteria: (I) systemic diseases such as hypertension, diabetes, cardiovascular system diseases, and acquired immune deficiency syndrome; (II) immunosuppressive agents or glucocorticoid therapy; (III) pregnancy or lactation; (IV) smoking habits; (V) other oral diseases, such as caries, fillings or crowns affecting the periodontal state at any sampling site; and (VI) the patients had received the periodontal treatment or taken the medicine that would affect the condition of the periodontal tissue or their immune systems in the past six months.

The protocol for collecting gingival samples was approved by the Medical Ethics Committee of Nanjing Stomatological Hospital, Medical School of Nanjing University, and the ethics approval number was 2016NL-010(KS). All experiments were performed in accordance with relevant guidelines and regulations. Participants were informed of the purpose of experiments and provided informed consent.

### Immunohistochemistry

The gingival tissue samples were soaked in 4% paraformaldehyde for 24 hours, embedded in wax and cut to 3 μm in thickness. After regular deparaffinization, rehydration and antigen retrieval with heated citrate buffer (pH 6.0), the sections were incubated with anti-MLKL, anti-MLKL (phospho S358), anti-RIPK3, anti-RIPK1 (Abcam, US) or anti-caspase8 (Proteintech, China) overnight at 4 °C. Then, the slides were washed three times with phosphate-buffered saline and incubated with secondary antibodies (MaxVision, China) at room temperature for 30 min. Diaminobenzidine (DAKO, USA) was used as a chromogenic agent to detect antibody binding.

### Semi-quantitative Analysis (SQA)

The scoring method described in H. Lucas^14^ was used for semi-quantitative evaluation. Two experienced “blinded” readers scored the slides according to the percentage of positive cells: 0 points implied less than 10% positive cells 1 point implied 11–25% positive cells, 2 points implied 26–50% positive cells, 3 points implied 51–75% positive cells, and 4 points implied greater than 75% positive cells.

### Quantitative PCR

Total RNA for real-time PCR was extracted from gingival samples using a QIAshredder and RNeasy Easy Mini kit (Qiagen, Hilden, Germany) and reverse transcribed into cDNA using a PrimeScript^TM^ RT reagent kit with gDNA Eraser (Perfect Real Time) (Takara, Japan). The actin gene was used as an internal control. The PCR primer sequences were as follows: RIPK1 5′-GGCATTGAAGAAAAATTTAGGC-3′ and 3′-TCACAACTGCATTTTCGTTTG-5′; RIPK3 5′-CTCTCTGCGAAAGGACCAAG-3′ and 3′-CATCGTAGCCCCACTTCCTA-5′; MLKL 5′-CTCTTTCCCCACCATTTGAA-3′ and 3′-TCATTCTCCAGCATGCTCAC-5′; Casp8 5′-AAGTGCCCAAACTTCACAGC-3′ and 3′-TACTGTGCAGTCATCGTGGG-5′; and ACTIN 5′-GTGGGGCGCCCCAGGCACCA-3′ and 3′-CGGTTGGCCTTGGGGTTCAGGGGGG-5′.

### Bacterial Strains

*P. gingivalis* ATCC 33277 was anaerobically (85% N2, 5% H2 and 10% CO2) grown at 37 °C in BHI medium with hemin (5 μg/mL), menadione (1 μg/mL) and yeast extract (1 μg/mL). The concentration of bacteria was 10^9^ CFU/mL when the optical density at 600 nm was 1. Viable *P. gingivalis* were obtained from the liquid medium by centrifugation. Bacterial pellets were washed three times using sterile phosphate-buffered saline (PBS) and resuspended in the cell culture medium without antibiotics.

### Cell Culture

HPDLFs were from 10 to 18-year-old patients who needed orthodontic extraction of healthy premolars. Patients and their parents were informed of the purpose of the experiment. Tissue explants obtained from the middle third of the root were cultured in DMEM (Gibco, USA) with 20% fetal bovine serum (Gibco, Australia) and 10% penicillin/streptomycin solution (Life Technologies). Cells at the third to sixth passage were used in the experiment. Necrostatin-1(Nec-1) (Selleckchem, USA), GSK’872 (Merck-Millipore, Germany) and necrosulfonamide (NSA) (Enzo Life Sciences, USA) were utilized to block RIPK1, RIPK3 and MLKL, respectively. The PDLFs were pretreated with these inhibitors for 2 hours and then stimulated with *P. gingivalis*.

THP-1 human monocytic cells (ATCC) were grown in RPMI 1640 medium (Gibco) with 10% fetal bovine serum (FBS, Gibco) and 1% penicillin/streptomycin solution. Cells were treated with *P. gingivalis* of different multiplicity of infection (MOI) and continued to be incubated at 37 °C, 5% CO2 under the normal oxygen condition in a humidified environment.

### Preparation of DAMPs

The DAMPs was prepared as previously described^36^. 10^7^ THP-1 cells or PDLFs cells were resuspended in 1 mL PBS. Cell suspension solution was exposed in −80 °C refrigerator directly for 30 min and then thawed at 37 °C for 30 min, repeating freezing and thawing for 5 times. The necrotic cell supernatant was achieved by centrifugation at 2000 rpm at 4 °C.

### SiRNA Transfection

Envirus^TM^ (Engreen Biosystem) was used to transfect PDLFs with small interfering RNA targeting RIPK1, RIPK3, or MLKL or a control siRNA with no target (GenePharma, China). The efficacy of knockdown was analyzed by Western blot 72 h after transfection.

### Western Blotting

The collected gingival tissues were immediately stored at −80 °C. The gingival tissues were cut into pieces, lysed with RIPA buffer and homogenized with a homogenizer. Cells were washed with PBS and lysed. The concentration of total proteins was determined using a Nanodrop (Thermo Fisher, USA). Equal amounts of protein were separated via 4–12% SDS-PAGE (Genscript, China) and transferred to a PVDF membrane. The membranes were blocked with 5% bovine albumin (Sigma, USA) and incubated with anti-MLKL, anti-MLKL (phospho S358), anti-RIPK1, anti-RIPK3, anti-RIPK3 (phosphor Ser227) (CST, USA), anti-cellular FLICE (FADD-like IL-1β-converting enzyme)-inhibitory protein (c-FLIP) (Sigma) or GAPDH (Bioworld), followed by secondary antibodies (Bioworld). The signals were detected using ImageQuant LAS 4000. The optical density of each lane was read using ImageJ (Bethesda, USA).

### ELISA and Cytotoxicity Assays

The LDH assay was performed according to the manufacturer’s instructions (Promega, USA). The levels of IL-6 or MCP-1 released by the hPDLFs were measured using an enzyme-linked immunosorbent assay (ELISA) kit (MultiSciences, China). The optical density was read using a SpectraMax M3 (Molecular Devices, Sunnyvale, CA, USA).

### Statistical analysis

The analysis was performed using GraphPad (GraphPad Software, Inc.). All data are presented as the mean ± standard deviation (SD) and were analyzed using the nonparametric Wilcoxon test or Student’s t-test. Values of P < 0.05 were considered statistically significant.

## Data Availability

All data generated or analysed during this study are included in this published article or available from the corresponding author on reasonable request.
